# Continuation of epidermal growth factor receptor tyrosine kinase inhibitor treatment prolongs disease control in non-small-cell lung cancers with acquired resistance to EGFR tyrosine kinase inhibitors

**DOI:** 10.18632/oncotarget.4570

**Published:** 2015-07-09

**Authors:** Qi Chen, Qi Quan, Lingyu Ding, Xiangchan Hong, Ningning Zhou, Ying Liang, Haiying Wu

**Affiliations:** ^1^ State Key Laboratory of Oncology in South China, Guangzhou, China; ^2^ Department of Oncology, Sun Yat-Sen University Cancer Center, Guangzhou, China; ^3^ Collaborative Innovation Center of Cancer Medicine, Sun Yat-Sen University Cancer Center, Guangzhou, China

**Keywords:** non-small-cell lung cancer, acquired resistance, continuation of epidermal growth factor receptor tyrosine kinase inhibitors, beyond PD, local treatment

## Abstract

**Objectives:**

Patients with non-small-cell lung cancer (NSCLC) develop acquired resistance to epidermal growth factor receptor tyrosine kinase inhibitors (EGFR TKIs) after tumor regression. No approved targeted therapies are currently available after initial EGFR TKI treatment. This study investigated the efficacy of continuing EGFR TKI therapy with local treatments for patients with NSCLC and local progression or minimal/slow progression on TKI therapy.

**Materials and Methods:**

Fifty-five patients with NSCLC treated with EGFR TKIs and developed acquired resistance to the drug were included. Initial response to target therapy, median progression free survival (PFS1), progression pattern, and first progression site were assessed. Median progression free survival to physician assessment progression (PFS2) and difference between PFS1 and PFS2 (PFS difference) were also recorded.

**Results and Conclusion:**

PFS1 was 11.2 months, PFS2 was 20.3 months, and PFS difference was 8.3 months. Nineteen patients (34.5%) who manifested progression received local therapy, and 16 (28.6%) underwent rebiopsy after progression with six positive EGFR T790M mutations detected. Cox proportional hazards regression model showed that only the first line of treatment was significantly correlated with PFS difference. NSCLC patients with acquired resistance to EGFR TKIs could benefit from the same TKI therapy through months to years of disease control.

## INTRODUCTION

Epidermal growth factor receptor tyrosine kinase inhibitors (EGFR TKIs) are the standard of care in EGFR-mutant non-small-cell lung cancer because of its superior efficacy over chemotherapy. [1, 2] However, patients who initially responded to EGFR-TKIs would eventually present a median of 10–14 months in response evaluation criteria in solid tumors progressive disease (RECIST PD), [3–5] which clinically manifests as tumor progression and symptomatic decline.

Acquired resistance to EGFR TKIs is caused by several molecular mechanisms, including the presence of the T790M missense mutation, MET amplification, and transformation to small-cell histology. [6] Currently, no approved targeted therapies or efficient treatments are available for patients upon progression.

We selected NSCLC patients with local progression or minimal/slow progression on TKI therapy in our study. There is ongoing benefit from the targeted therapy in other sites of (non-progressing) disease due to continuing suppression of sensitive clones that have not yet developed acquired resistance. Consistent with this, patients with EGFR-MT disease who progress often experience a disease flare when the EGFR-TKI is discontinued, and re-challenge of these patients with the same EGFR-TKI after only a short time off therapy can lead to re-responses. [7–9] In addition, treatment beyond progression of EGFR-MT NSCLC with an EGFR-TKI has been associated with improved overall survival, compared to those in whom the TKI was permanently discontinued. [10] Analogous benefits of continuation of trastuzumab beyond progression have been well described in metastatic breast cancer. [11]

This clinical phenomenon has impelled physicians to continue TKI therapy beyond progression on EGFR TKI agents. Ongoing prospective studies evaluate strategies of continuation of erlotinib beyond RECIST progression (ASPIRATION) [12] and gefitinib combined with chemotherapy beyond RECIST progression (IMPRESS); [13] nevertheless, results remain inconclusive. Therefore, we designed this study to investigate the efficacy and safety of continuation of EGFR TKI therapy with necessary local treatments for NSCLC patients with local progression or minimal/slow progression on TKI therapy. The potential factors that affect the effectiveness of this strategy were also discussed.

## RESULTS

### Patient characteristics

**Figure 1:**
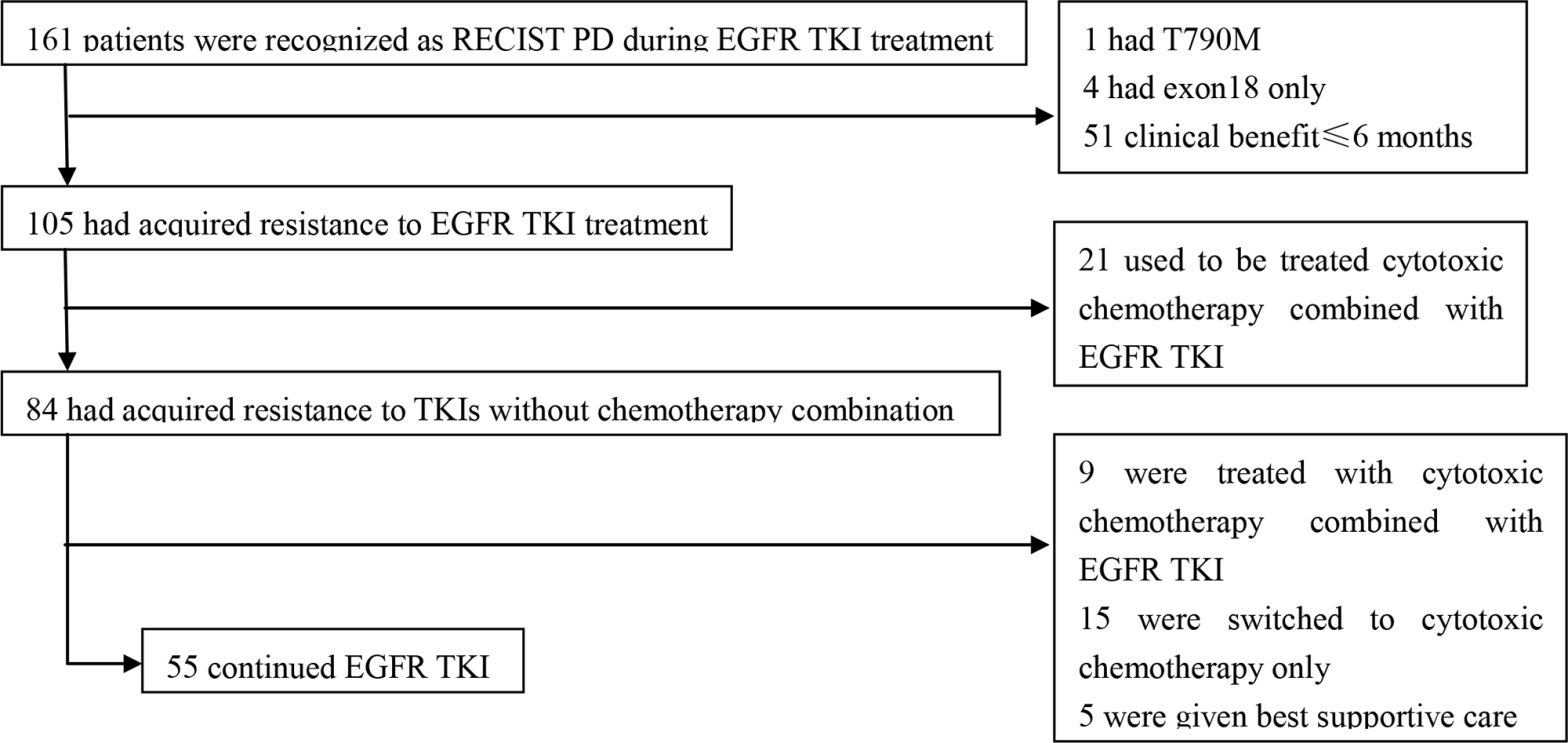
Flowchart of patient selection EGFR, epidermal growth factor receptor; TKI, tyrosine kinase inhibitor; RECIST, Response Evaluation Criteria in Solid Tumors; PD, progression disease.

From December 2010 to January 2015, patients from the Sun Yat-Sen University Cancer Center with histologically confirmed stage IIIB/IV NSCLC were included in this study. We initially screened 261 patients (Figure 1) with RECIST progression on EGFR TKIs, and 55 patients who satisfied the inclusion criteria were eventually analyzed. The clinical and molecular baseline characteristics of the 55 patients are shown in Table 1. The patients were composed of 23 men and 32 women, and most of them were non-smokers (44, 80%). The tumor pathology of the patients was all adenocarcinoma, except for two squamous cell carcinomas. Seventeen patients were older than 60 years. Forty-three cases (78.2%) harbored EGFR-sensitive mutations (including 23 exon 19 deletions and 20 exon 21 L858R mutations), four cases (7.3%) with wild type, and eight cases with unknown mutational status (14.5%). As shown in Table 1, lung (44/55, 80%), brain (4/55, 7.3%), and bone (4/55, 7.3%) were the leading sites of progression. A patient simultaneously developed brain and lung progression, and five patients successively developed progression in the two sites. As for local treatments (19/55, 34.5%) upon progression, four patients (4/19, 21.1%) received lung radiofrequency ablation after pulmonary progression, one patient (1/19, 5.3%) received lung stereotactic radiotherapy, and four (4/19, 26.3%) patients with pleural effusion progression received ultrasound-guided drainage plus bleomycin injection to the thoracic cavity. In patients with CNS progression, four received whole brain radiation therapy (WBRT), one received gamma knife radiosurgery, one sequentially received WBRT and gamma knife radiosurgery, and one received brain metastases resection surgery and WBRT surgery. Three of four patients (3/4, 75%) with bone metastasis progression continued EGFR TKI treatment with bone radiation therapy.

**Table 1:** Patients characteristics (*n* = 55)

	*N*	%
**Age(y)**		
**Median (range)**	55(31–77)	
**Gender**		
**Female**	32	58.2
**Male**	23	41.8
**Smoking history**		
**Never-Smoker**	44	80.0
**Former/smoker**	11	20.0
**Pathology**		
**Adenocarcinoma**	53	96.4
**squamous cell carcinoma**	2	3.6
**EGFR mutation status**		
**Exon 19 deletion**	23	41.8
**Exon 21 L858R**	20	36.4
**Wild type**	4	7.3
**Unknown**	8	14.5
**Disease stage**		
**Stage IV**	43	78.2
**Recurrent**	12	21.8
**Initiation of EGFR-TKI**		
**1st line**	30	54.5
**2/3rd line**	25	45.5
**TKI regimen choice**		
**Erlotinib**	37	67.3
**Gefitinib/Icotinib**	18	32.7
**Local therapy**		
**No**	36	65.5
**Yes**	19	34.5
**Site of RECIST PD**		
**Lung**	44	80.0
**Bone**	4	7.3
**Brain**	4	7.3
**Lymph node**	1	1.8
**Visceral (liver, adrenal gland)**	2	3.6
**Progression type**		
**Local progression**	10	18.2
**Slow/Minimal progression**	45	81.8

EGFR, epidermal growth factor receptor; TKI, tyrosine kinase inhibitor; RECIST, Response Evaluation Criteria in Solid Tumors; PD, progression disease.

Sixteen patients (28.6%) had rebiopsy after resistance at the following sites: pleural effusion (6/16, 37.5%), lung (5/16, 31.3%), supraclavicular lymph nodes (3/16, 18.8%), ascetic fluid (1/16, 6.3%), and brain metastases (1/16, 6.3%). Of 16 patients, 14 (87.5%) had their rebiopsy tissue examined by molecular tests (Table 2), three patients underwent EGFR mutation and MET amplification tests, and 11 patients had EGFR mutation tests. Among these 14 EGFR mutation tests samples, we detected six (42.9%) positive EGFR T790M mutations with one sensitive mutation and two (14.3%) wild types. For the two wild-type rebiopsy patients, their rebiopsy sites were both in the lung. One patient harbored EGFR mutation of L858R in exon 21 in lung biopsy tissues, and the other patient had wild-type EGFR in brain metastasis tissues; TKI therapy resulted in stable disease in the lung for 8.25 months. One (33.3%) positive result was detected in the MET amplification tests, and the rebiopsy sample of this patient harbored the T790M mutation.

**Table 2:** EGFR mutation status before and after RECIST PD

patient	first biopsy site	EGFR mutation status before RECIST PD	rebiopsy site	EGFR mutation status after RECIST PD
1	lung	exon 19 deletion	hydrothorax	exon 19 deletion
2	lung	exon 19 deletion	ascites	exon 19 deletion
3	lung	unkonwn	hydrothorax	exon 19 deletion
4	lung	L858R	supraclavicular lymph nodes	L858R
5	brain	wild type	lung	wild type
6	supraclavicular lymph nodes	exon 19 deletion	lung	exon 19 deletion+T790M mutation
7	hydrothorax	L858R	hydrothorax	L858R
8	lung	exon 19 deletion	supraclavicular lymph nodes	exon 19 deletion+T790M mutation
9	lung	L858R	hydrothorax	L858R+T790M mutation
10	lung	exon 19 deletion	lung	exon 19 deletion+T790M mutation
11	supraclavicular lymph nodes	exon 19 deletion	supraclavicular lymph nodes	exon 19 deletion
12	lung	L858R	lung	wild type
13	lung	exon 19 deletion	hydrothorax	exon 19 deletion+T790M mutation
14	lung	exon 19 deletion	lung	exon 19 deletion, T790M mutation, MET amplification(+)

EGFR, epidermal growth factor receptor; RECIST, Response Evaluation Criteria in Solid Tumors; PD, progression disease

### Survival data

Data cut-off date for follow-up was January 23, 2015, and the median follow-up duration was 20.93 months from the initial TKI therapy to physician assessment PD (range: 8.51–81.87 months). At the time of the data cut off, 23 patients (41.1%) exhibited physician assessment PD and 11 (19.6%) died. The median progression free survival (PFS1) was 11.2 months (95% CI, 9.4–14.7 months), the median PFS2 was 20.3 months (95% CI, 17.1–24.1 months; Table 3), and the median PFS difference was 8.3 months (95% CI, 6.4–10.2 months; Table 3). Hence, 80% of patients can further benefit from continuation of EGFR TKI treatment for more than 3 months. Moreover, disease control persisted for more than 12 months in 26% of patients without significant clinical progression.

**Table 3:** Survival data

PFS1	
Median(95%CI)	11.2(9.4–14.7)
PFS2	
Median(95%CI)	20.3(17.1–24.1)
PFS Difference	
Median(95%CI)	8.3(6.4–10.2)

PFS, progression free survival; CI, confidence interval.

Smoking history, gender, age, pathology, initial TKI efficacy, TKI regimen, first line of treatment, disease stage, and EGFR mutation status were used as variables in univariate analysis. The results demonstrated that female patients, patients who never smoked, and patients who had not undergone chemotherapy before a TKI treatment presented high possibility of achieving effective outcomes with continued EGFR TKI monotherapy (Table 4).

**Table 4:** Survival analysis of continued TKI

*P*	Univariate analysis			Multivariate analysis		
	PFS1	PFS2	PFSDifference	PFSDifference	Hazard ratio[95%CI]	
**gender**	0.206	0.136	0.164			
**Age (>60 y vs. <60 y)**	0.367	0.722	0.855			
**Smoking history**	0.231	**0.026**	**0.031**			
**Pathology**	**0.004**	**0.024**	0.321			
**EGFR mutation status**	0.663	0.646	0.785			
**Disease status at initiation of TKI(Stage IV vs. Recurrent)**	0.417	0.112	0.078			
**Disease stage**	0.061	0.353	0.542			
**TKI regimen choice**	**0.002**	0.111	0.332			
**Local therapy**	/	0.932	0.889			
**Best response to TKI**	0.507	0.279	0.402			
**Initiation of EGFR-TKI(2/3rd line vs. 1st line)**	**0.023**	0.248	**0.035**	**0.048**	2.192(2/3rd line vs. 1st line) [1.008–4.768]	

EGFR, epidermal growth factor receptor; TKI, tyrosine kinase inhibitor; PFS, progression free survival; CI, confidence interval.

The first line of treatment was significantly correlated with PFS difference (Figure 1) under multivariate Cox proportional hazards regression model (hazard ratio [HR] for chemotherapy vs. TKI, 2.192; 95% CI, 1.008–4.768, *P* = 0.0048; Table 4)

The most common adverse event was grade 1 or 2 rash, which affected seven patients (12.7%), whereas no grade 3 skin rash was observed. Moreover, no dose reduction or discontinuation of TKI caused by unbearable TKI-associated toxicity was required.

## DISCUSSION

Patients who developed local or slow/minimal progression (oligoprogression) after EGFR TKI treatments present unique clinical characteristics. As no approved targeted therapies are currently available for patients with acquired resistance, they choose between standard cytotoxic chemotherapy with or without EGFR TKI continuation or enroll in clinical trials. In this study, continuation of the same EGFR TKI therapy in addition to necessary local therapy (including radiation, ultrasound-guided drainage plus bleomycin injection to thoracic cavity, and surgery) is correlated with a median time to physician assessment progression of 21 months, thus extending disease control by more than 9 months after RECIST progression. The median time to progression in groups choosing pemetrexed plus platinum chemotherapy after prior EGFR TKI treatment failure was 6.1 months. [14] Several factors contributed to the efficacy of the treatment in patients with NSCLS with acquired resistance to EGFR TKI (local or slow/minimal progression); such factors include special clinical course of acquired resistance disease, continuation of TKI therapy for sensitive tumor cells, and potential benefits of local treatment.

Few articles reported the outcomes of continued EGFR TKI for patients with acquired resistance to the targeted therapy. According to Jackman's definition, [15] patients with acquired resistance to EGFR TKIs were classified under a unique patient population. These patients had improved outcomes with continuous EGFR TKI therapy. Moreover, approximately 80% of the patients harbored a drug sensitivity-associated EGFR mutation site and presented improved surgical outcomes with cytotoxic chemotherapy. [5, 16] Even with the development of acquired resistance, these patients with local progression or minimal/slow progression on TKI therapy resulted in long survival, particularly those with the emergence of the T790M mutation, which is correlated with improved beyond-progression outcomes. [17]

All patients in this study continued the same EGFR TKI treatment after progression, which probably contributed to their effective clinical outcomes. A previous study indicated that during the development of acquired resistance to EGFR TKIs, all cells remained oncogene addicted; the most common etiology of acquired resistance was the presence of the T790M mutation in few cells, which were only a small fraction of total alleles, and most cells remained sensitive. [18] This theory could partly explain the effectiveness of TKI therapy after acquired resistance. Moreover, non-stop targeted therapy prevented potential disease flare, which has been reported in patients who discontinued erlotinib or gefitinib after developing acquired resistance. [8, 9]

In 2010, a clinical definition of acquired resistance to EGFR-TKIs in NSCLC [15] was proposed for those who responded (≥ 6 months) to initial gefitinib or erlotinib treatment with a drug sensitivity-associated mutation site or objective clinical benefit from treatment with an EGFR TKI. Patients with local or minimal/slow progression to EGFR TKI benefited from continuous targeted treatment. The established clinical definition is reasonable as confirmed in the present research, in which patients with several characteristics exhibited a prolonged PFS of 8.3 months. Moreover, long PFS1 resulted in high PFS difference, which is consistent with the acquired resistance definition. The only significant factor affecting the PFS difference in multivariate Cox proportional hazards regression model is the first line of treatment. Thus, patients who did not receive chemotherapy before EGFR-TKIs could present a high PFS difference. Hence, patients who received chemotherapy before EGFR TKI therapy exhibit poor performance at the initiation of targeted therapy, resulting in low PFS1 and PFS differences.

Genomic analysis comparison of rebiopsy and primary tumor samples is shown in Table 4. In 14 patients who underwent rebiopsy and T790M mutation test after resistance, the frequency of EGFR T790M mutation was 42.9%, which is consistent with a previous report. [17] One (33.3%) positive MET amplification case was found in three tested samples, which could be attributed to limited test cases. No small-cell histologic transformation was detected in the rebiopsy tumor samples. Rebiopsy after development of acquired resistance and genomic analysis of progression sites should be included in routine work because they may provide useful information for tailoring subsequent treatment strategies.

In conclusion, this study showed that continuation of EGFR TKI therapy with necessary local therapy or treatment can be used as a management option for patients who developed oligoprogression during EGFR TKI therapy. Patients with acquired resistance to EGFR TKIs presented a unique clinical course and could benefit from continuation of EGFR TKI treatment, resulting in months to years of disease control and tolerance. However, this study was limited by the small number of enrolled patients and inconclusive overall survival data. A prospective multicenter evaluation of continuation of EGFR-TKI treatment must be performed on patients who developed local or minimal/slow progression according to the type of resistance mechanisms.

## PATIENTS AND METHOD

### Patient eligibility

Patients with lung cancer who developed acquired resistance to EGFR TKI with documented slow or local progression after TKI therapy and would continue single-agent EGFR TKI until physician assessment PD were enrolled in this study. Physician assessment PD was defined as symptomatic progression and/or multiple progression (≥ four sites of extracranial progression) and/or vital organ progression. Patients may undergo necessary local therapy or treatment (radiation therapy, radiofrequency ablation, gamma knife radiosurgery, or ultrasound-guided drainage plus bleomycin injection to the thoracic cavity) for a site of progressive disease.

As some patients currently do not have their tumor EGFR mutation status determined before starting on EGFR TKI, the following criteria were used to screen patients who may benefit from the continuation of TKI according to the Jackman's definition of acquired resistance: [15]

- A tumor that harbors an EGFR mutation known to be associated with drug sensitivity (i.e., G719X, exon 19 deletions, L858R, and L861Q).
- Objective clinical benefit from treatment with an EGFR TKI as defined by either of the following:
  - Documented partial or complete response (RECIST) or
  - Significant and enduring (≥6 months) clinical benefit (stable disease as defined by RECIST) after initiation of first generation EGFR TKI.

Clinical characteristics and treatment courses, including tumor EGFR mutation status and rebiopsy results if identified, were reviewed from electronic medical records of all subjects to determine the mechanism of acquired resistance. Outcomes of interest included time to RECIST PD, time to physician assessment PD, and overall survival from time of acquired resistance. Nonsmokers were defined as those who had smoked <100 cigarettes in their lifetime.

### Treatment

All patients enrolled were orally given 150 mg of erlotinib daily, 250 mg of gefitinib daily, or 125 mg of icotinib t.i.d. The patients continued treatment beyond RECIST PD until physician assessment PD, death, or unacceptable toxicity was reached, whichever came first. Patients continued oral TKI therapy during local therapy intervals.

### Response assessment and toxicity evaluation

In our institute, a RECIST evaluating committee comprising experienced radiologists evaluated tumor shrinkage or progression. The date of progression was defined based on routine surveillance imaging (every 2 to 3 months) and/or symptomatic progression leading to earlier radiographic evaluation using the version of RECIST 1.1. Adverse events were graded according to the modified 4.0 version of the National Cancer Institute Common Toxicity Criteria.

### Statistical analysis

Initial PFS1 was defined as the interval between the beginning of EGFR-TKI and the RECIST progression time. PFS2 was defined from the start of TKI treatment to the date at which physician assessment progression or death was noted. PFS difference was defined as the difference between PFS1 and PFS2. PFS was analyzed by Kaplan–Meier method, and log-rank test was used to compare the difference within different groups. Multivariate Cox proportional hazards regression model was used to evaluate independent predictive factors associated with PFS difference. A two-sided *P* value of less than 0.05 was considered statistically significant. All analyses were conducted using SPSS software version 11.0 for Windows.
